# A Comparative Study of Accident Risk Related to Speech-Based and Handheld Texting during a Sudden Braking Event in Urban Road Environments

**DOI:** 10.3390/ijerph17165675

**Published:** 2020-08-06

**Authors:** Rui Fu, Yunxing Chen, Qingjin Xu, Yuxi Guo, Wei Yuan

**Affiliations:** 1 School of Automobile, Chang’an University, Xi’an 710064, China; furui@chd.edu.cn (R.F.); qingjinxu@outlook.com (Q.X.); yxguo@chd.edu.cn (Y.G.); yuanwei@chd.edu.cn (W.Y.); 2 School of Mechanical Engineering, Hubei University of Arts and Science, Xiangyang 441053, China

**Keywords:** speech-based texting, handheld texting, brake reaction time, rear-end accident probability, driving behavior characteristics, lead vehicle deceleration rate

## Abstract

The use of mobile phones while driving is a very common phenomenon that has become one of the main causes of traffic accidents. Many studies on the effects of mobile phone use on accident risk have focused on conversation and texting; however, few studies have directly compared the impacts of speech-based texting and handheld texting on accident risk, especially during sudden braking events. This study aims to statistically model and quantify the effects of potential factors on accident risk associated with a sudden braking event in terms of the driving behavior characteristics of young drivers, the behavior of the lead vehicle (LV), and mobile phone distraction tasks (i.e., both speech-based and handheld texting). For this purpose, a total of fifty-five licensed young drivers completed a driving simulator experiment in a Chinese urban road environment under five driving conditions: baseline (no phone use), simple speech-based texting, complex speech-based texting, simple handheld texting, and complex handheld texting. Generalized linear mixed models were developed for the brake reaction time and rear-end accident probability during the sudden braking events. The results showed that handheld texting tasks led to a delayed response to the sudden braking events as compared to the baseline. However, speech-based texting tasks did not slow down the response. Moreover, drivers responded faster when the initial time headway was shorter, when the initial speed was higher, or when the LV deceleration rate was greater. The rear-end accident probability respectively increased by 2.41 and 2.77 times in the presence of simple and complex handheld texting while driving. Surprisingly, the effects of speech-based texting tasks were not significant, but the accident risk increased if drivers drove the vehicle with a shorter initial time headway or a higher LV deceleration rate. In summary, these findings suggest that the effects of mobile phone distraction tasks, driving behavior characteristics, and the behavior of the LV should be taken into consideration when developing algorithms for forward collision warning systems.

## 1. Introduction

According to statistics from the World Health Organization, road traffic crashes cause approximately 1.35 million deaths worldwide and 20 to 50 million non-fatal injuries each year [1]. Among all traffic accidents, rear-end collisions are one of the most common types, respectively accounting for about 20% and 18% of all traffic crashes in Shanghai, China, [2] and the United States [3]. As smartphone devices are becoming used more frequently, the use of a mobile phone while driving has been identified as one of the main contributing factors to rear-end accidents [4,5]. This has also led legislative bodies in many countries to ban the use of mobile phones while driving, but the phenomenon remains very widespread [6,7,8]. Thus, to reduce the number of rear-end accidents, the development of distraction countermeasures, such as forward collision warning systems, is considered one of the most promising ways to reduce the potential impact of mobile phone distraction tasks [9,10]. To develop an effective forward collision warning system, it is crucial to accurately predict potential risk, which depends on quantifying the factors that cause a rear-end accident during mobile phone distracted driving. Therefore, in-depth study of the risk assessment parameters and related influencing factors of rear-end accident risk when drivers use mobile phones is crucial for the development of effective countermeasures to reduce rear-end accident risk and severity.

### 1.1. Accident Risk Evaluation during a Sudden Dangerous Event

A driver’s brake reaction time (BRT) is one of the frequently used surrogate measures for the evaluation of the accident risk during a sudden braking event [10,11]. The BRT is defined as the time from when the brake light of the lead vehicle (LV) turns on to when the subject vehicle starts to brake. Many scholars’ research on the effects of mobile phone use on BRT has mainly focused on mobile phone conversations and handheld texting [10,12,13,14]. Numerous studies have shown that using a mobile phone while driving increases the driver’s reaction time [10,14]. Comparative studies of the effects of handheld and hands-free phone use on driver reaction time have been conducted by many scholars [10,12,15]. He et al. [16] and Yager [17] studied the effects of handheld and speech-based texting on BRT and indicated that handheld texting had a greater impact than speech-based texting.

The probability of an accident is also an important evaluation indicator by which to estimate the increased accident risk caused by mobile phone distraction tasks [9,14,18]. Many researchers have examined the impacts of mobile phone conversations and handheld texting on accident risk and found that handheld texting while driving is more dangerous than engaging in phone conversations [13,19]. Many research results have indicated that texting while driving considerably increases the risk of accidents [1,14,18,20,21]. 

### 1.2. Potential Factors Influencing Accident Risk during a Sudden Dangerous Event 

Car-following is a complex driving behavior in daily driving activities. Drivers must continuously pay attention to the surrounding road traffic conditions, especially the driving state of the LV. To ensure driving safety in the car-following scene, the driver should negotiate the car-following speed (i.e., the speed of the subject vehicle during car-following) and car-following distance (i.e., the distance between the subject vehicle and the LV) according to the driving status of the LV [22]. Choudhary and Velaga [9] designed a sudden braking event so that the LV would suddenly stop when the distance between the LV and the subject vehicle was reduced to a fixed value of 40 m and studied the effect of driver’s reaction time and approach speed on accident probability during mobile phone distracted driving conditions (both conversation and texting). However, in a real-world driving environment, the driver’s car-following speed and car-following distance are constantly changing. Hence, the time headway (i.e., the ratio of the car-following distance to the speed of the subject vehicle) could be more influential than the speed with regard to rear-end accident risk, because the time headway takes into account both the speed and the car-following distance. Winsum and Heino [23] indicated that rear-end accidents are often due to insufficient time headway, delayed brake response, and insufficient brake force. A number of studies have focused on assessing the probability of accidents associated with mobile phone distracted driving conditions, but very few have considered the effects of driving behavior characteristics (e.g., the initial time headway and BRT) and the behavior of the LV (e.g., the LV deceleration rate).

Time headway is considered to be an important factor related to rear-end accident probability [23,24,25]. However, it has been found that the initial time headway (ITHW; the time headway at the LV brake onset) can better predict the rear-end accident risk than the mean time headway in car-following situations. This is because the mean time headway may be different from the time headway when the LV suddenly brakes; thus, the ITHW can better reflect the level of situational urgency and directly affect the rear-end accident risk [2,24]. Many previous studies have investigated the impact of mobile phone distraction on driving performance in terms of the time headway [26,27,28], whereas very little research has been conducted on the effect of the time headway on accident probability during mobile phone distracted driving, and even fewer studies have examined the effect of the ITHW. In addition, the BRT is also regarded as an important factor associated with rear-end accident risk [10,23]; however, it is unclear from the existing studies how the BRT affects rear-end accident probability. Previous studies have suggested that the behavior of the LV (e.g., the LV deceleration rate) has a very important influence on the driving performance of the subject vehicle [2,29]. Nevertheless, the effects of the behavior of the LV (e.g., the LV deceleration rate) on the rear-end accident probability in car-following situations have not been well studied. 

The influences of driver demographic factors like age and gender on accident risk during sudden dangerous events under mobile phone distracted driving conditions have been studied by many scholars [10,14,19,20,21]. Young and inexperienced drivers have a higher accident risk than older and experienced drivers while engaged in mobile phone distraction tasks during driving [21]. Li et al. [10] tested the effects of gender on risk and indicated that male drivers were more likely to be involved in an accident during mobile phone distracted driving than female drivers. In recent years, the influences of factors related to mobile phone usage habits on accident risk have also been studied by several researchers [19,30]. In addition, the road environment also plays an important role in traffic accidents. Some studies have found that there is a higher risk of using mobile phones while driving on urban roads than on rural roads [14,31]. Moreover, the use of mobile phones during driving on urban roads is more frequent than on rural roads [32].

### 1.3. Impact of Mobile Phone Distraction on Accident Risk during a Sudden Dangerous Event

The use of mobile phones while driving is regarded as a major factor that affects the risk of accidents, especially during sudden dangerous events [10,19,33]. Mobile phone use while driving is particularly prevalent among young drivers and significantly increases the risk of accidents [8,30,34]. The use of mobile phones during driving may be more dangerous for these young drivers due to them having less driving experience. In addition to mobile phone conversations, texting while driving is becoming more prevalent [8,35,36]. It has also been found that young drivers are more willing to text during driving because it is their main means of social communication [8,37]. Additionally, drivers are more likely to be involved in accidents, and the risk is increased by many times when they are engaged in texting [14,19,20,38]. Young drivers can also cause more serious accident risks [8,20]. Hence, it is very important to investigate the impact of texting while driving on the driving safety of young drivers.

To date, most studies on the impact of mobile phone use on accident risk have been primarily focused on mobile phone conversations and handheld texting [10,12,15,19], and there is very little existing research on the impact of speech-based texting on the risk of rear-end accidents during car-following [16]. Existing research on the impact of speech-based texting has mainly been conducted in developed countries, such as the UK, Canada, and the USA. Voice messages are sent mainly through Siri or Vlingo programs; both Siri and Vlingo send voice messages by converting voice messages into text messages and then sending them out [17]. In addition, Siri can read text messages but Vlingo cannot [17]. However, in China, it is very common for drivers to use WeChat software to send or receive speech-based texting during driving. The difference from foreign countries is that WeChat software sends and receives voice messages as a recording, and you can listen to the voice messages by clicking the recording icon [11]. Moreover, the WeChat software interface, length of speech-based texting, language system, and mobile phone usage habits are also different from those in developed English-speaking countries [11]. Therefore, it is necessary to examine the effects of the use of speech-based texting via WeChat software on accident risk in China, particularly in the case of a sudden braking event of the LV.

### 1.4. Research Gap

From the literature review presented in the preceding subsection, it is evident that most of the existing research on the effects of different types of mobile phone use on accident risk during a sudden braking event has focused on phone conversations and handheld texting tasks [9,10,15]. However, very few scholars have studied the impact of speech-based texting on rear-end accident risk in urban road environments. Moreover, a comparison of accident risk associated with speech-based texting and handheld texting (each with two difficulty levels), especially during sudden braking events of the LV, has not been examined. Another research limitation is that the quantitative effects of the driving behavior characteristics of young drivers (e.g., the initial time headway and BRT) and the behavior of the LV (e.g., the LV deceleration rate) on rear-end accident risk during mobile phone distracted driving have not been thoroughly studied.

To address the research gaps mentioned above, the main purpose of the present study is to identify potential predictors of rear-end accidents during sudden braking events in terms of driving behavior characteristics of young drivers, the behavior of the LV, and mobile phone distraction. For this purpose, the generalized linear mixed model (GLMM) method is used to statistically model the BRT and rear-end accident probability. Some specific contents of this study mainly focus on the following aspects: (1) quantifying the effects of mobile phone distraction on accident risk during sudden braking events in the Chinese urban road environment, (2) considering both speech-based and handheld texting tasks with two levels of difficulty, (3) quantifying the impacts of the ITHW, BRT, and LV deceleration rate on rear-end accident risk, (4) analyzing the accident risk associated with all factors (i.e., driver demographics, driving history, and mobile phone use habits).

## 2. Methodology

### 2.1. Participants

In total, 56 young participants aged between 21 and 30 years with valid driving licenses were recruited through online advertising. All recruited participants had normal vision or corrected to normal vision and had a good health condition. One participant failed to complete the experiment because of simulator sickness. The ethics protocol of the study was approved by the Ethics Committee of Chang’an University. A questionnaire was prepared to collect basic information about each participant, including the driver’s demographics, driving history, and mobile phone use habits. Table 1 presents the descriptive statistics of all participants’ basic information. The average age of the participants was 25.13 years old (SD = 2.57), and the sample group was composed of 40 males and 15 females. In 2014, Chinese female drivers accounted for 23.48% of all drivers, so our male to female ratio is consistent with the current gender distribution of drivers in road traffic [2]. The average driving experience was 3.05 (SD = 2.31) years. About 78% of the participants had a total mileage of less than 5000 km, 10.91% of the participants had driven between 5000 km and 10,000 km, and the remaining 10.91% had driven more than 10,000 km. In the past three years, 94.54% of the participants had not experienced a traffic accident, the percentage of those who had experienced only one traffic accident was 3.64%, and only one participant had experienced more than one traffic accident. None of the participants had been given traffic fines or experienced accidents due to the use of mobile phones. The statistical description of the mobile phone use habits while driving of all the participants revealed that most of them were more inclined to receive and send speech-based texting via WeChat software as compared to handheld texting. About 41.82% of the participants sometimes used speech-based texting, and 21.82% of them frequently used it. About 38.18% of the participants sometimes used handheld texting, and only 3.64% often used it.

### 2.2. Apparatus

**Figure 1:**
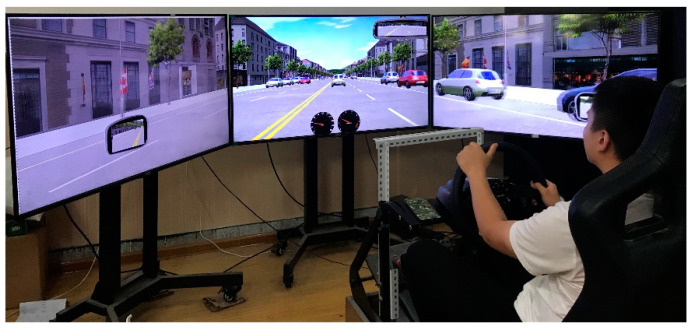
The driving simulator.

A fixed-base driving simulator was used for the study (see Figure 1) and included a steering wheel, gear selector (with automatic transmission), accelerator, and brake pedals. The driving scene was displayed on three 55-inch HD screens that provided a horizontal view of 120° at a 1920 × 1080 resolution refreshed at 60 Hz. Additionally, engine and road noises were simulated by a sound system. The computer system of the driving simulator recorded the following distance, subject vehicle (SV) speed, and deceleration data at a sampling frequency of 60 Hz.

### 2.3. Scenarios Design

A 3-km-long typical Chinese city road scene was created in the driving simulator. It was a two-way, four-lane road (each lane was 3.5 m wide) with a speed limit of 60 km/h and two non-motorized lanes. Before the formal experiment, the experimenter informed the participants that the speed limit of the test road was 60 km/h. To simulate the real environment of urban roads, there was moderate traffic flow in the opposite lane. There were only two vehicles (the SV and LV) in the driving direction. The LV stopped at a distance of 50 m from SV at the beginning of the scene. When the SV gradually approached the LV, it triggered the LV to travel to 50 km/h with an acceleration of 1 m/s^2^, and then maintained this constant speed. Each participant had to perform five different driving tasks: baseline (no phone use), simple speech-based texting, complex speech-based texting, simple handheld texting, and complex handheld texting.

**Figure 2:**
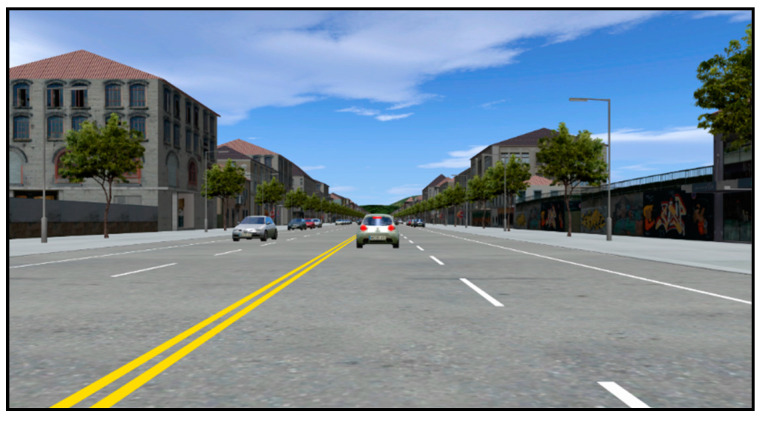
The lead vehicle (LV) sudden braking scenario.

During each experimental run, the participant drove the SV from the starting point and followed the LV with his or her real driving habits. During each mobile phone distracted driving event, the participant used a mobile phone from the beginning of the driving simulation experiment to the end. To simulate different environmental emergency levels, the LV was programmed to make three unexpected full stops with its brake lights on under each driving condition (see Figure 2). The three deceleration rates of the LV were 3, 5, and 8 m/s^2^ and the order was random [2]. To avoid rear-end collisions, the participants adopted braking behavior according to their own driving habits. When the rear-end collision avoidance behavior ended, The LV accelerated again from 0 to 50 km/h at an acceleration rate of 1 m/s^2^. Participants then continued to drive the SV to follow the LV until the three rear-end collision avoidance actions were completed. The experimenter recorded the accident data during each experiment. A total of 84 rear-end accidents occurred during the experiment, among which the numbers of the baseline (no phone use), simple speech-based texting, complex speech-based texting, simple handheld texting, and complex handheld texting conditions were 11, 16, 16, 20, and 21, respectively.

### 2.4. Secondary Task Design

Speech-based texting and handheld texting were considered as the secondary tasks in the study and were designed to include both simple and complex levels of difficulty. The simple tasks were single-digit addition and subtraction within 10, and the complex tasks were double-digit addition and subtraction within 100 [11,22,24]. WeChat software was used to send and receive messages between the experimenter and the participant during the secondary tasks. The participant’s mobile phone was always on the WeChat interface to communicate with the experimenter during mobile phone distracted driving. When participants listened and sent speech-based text messages, their attention could continue to focus on the road ahead. Therefore, compared with hand-held texting tasks, speech-based texting tasks involved less visual distraction. In addition, when the experimenter sent a speech-based text to the participant, the participant responded only by sending a speech-based text. The sending and receiving of handheld texts between the experimenter and the participant were similar.

### 2.5. Experimental Procedure

Upon reaching the driving simulator, each participant was introduced to the experimental requirements and asked to read and sign the informed consent form. They then completed a questionnaire, the detailed information of which is shown in Table 1. Before performing the formal experiment, each participant was asked to practice on the driving simulator for about 5–10 min to become familiar with it. Participants practiced driving simulator operations, including acceleration, deceleration, and steering, in a car-following scenario.

During the formal experiment, each participant was required to perform five driving tasks, namely one non-distracted driving task and four distracted driving tasks with different mobile phone use (simple and complex speech-based texting and simple and complex handheld texting). The participants used their personal phones for the distraction tasks. The experimenter sat in the room away from the driving simulator and sent WeChat messages to the participants, who were asked to reply to the messages using WeChat software as soon as possible after receiving them. The experimenter then sent the next message immediately, and the communication continued until the driving task was over. To avoid the effects of driving fatigue caused by long-term driving on the experimental results, the participants took at least 5 min of rest between two driving tasks. Moreover, to reduce the impact of learning effects on the experimental results, the driving tasks across participants and the LV deceleration rate were in random order. Furthermore, the participants were required to follow the LV with their own real driving habits and to obey the traffic rules and speed limits. In addition, the participants were told that if they felt any discomfort during the test, the experiment could be ended at any time. The test time for each participant to complete five driving tasks was approximately 30 min.

### 2.6. Analysis

#### 2.6.1. Dependent and Independent Variables

This study analyzed the effects of mobile phone distraction tasks on BRT and rear-end accident risk under different LV deceleration rates in a car-following scenario. The dependent variables were the BRT and rear-end accident probability. The driving conditions, driver demographics, driving history, mobile phone use habits, and LV deceleration rate were considered to be the independent variables of the BRT model and the rear-end accident probability model. The driving conditions included a baseline (no phone use), simple speech-based texting, complex speech-based texting, simple handheld texting, and complex handheld texting. Additionally, Table 1 presents the statistical details of the other independent variables, including the driving demographics (i.e., age and gender), driving history (i.e., years of driving, kilometers driven, traffic fines due to mobile phone use in the last three years, crash involvement history in the last three years, and traffic accidents due to mobile phone use in the last three years), and mobile phone use habits (i.e., frequency of speech-based texting while driving and frequency of handheld texting while driving). Three LV deceleration rates (i.e., 3, 5, and 8 m/s^2^) were considered for both models. The confidence level used in the models of the two dependent variables was 90%, i.e., the significance level of 0.1 (i.e., the *p*-value) was considered the standard of all variables in the models.

#### 2.6.2. Statistical Approach

The experimental data in this study may have had unobserved heterogeneity due to repeated observations from the same driver. To overcome this problem, GLMMs were developed to model the BRT and rear-end accident probability during the LV sudden braking scenario, as the GLMM approach can explain the possible heterogeneity via random effects [39,40]. The GLMM for the BRT was developed by using an identity link function. Because the rear-end accident probability was a binary variable (1 if an accident occurred and 0 otherwise), a GLMM with a logit link function was used for the rear-end accident probability. The odds ratio (OR) is estimated from the rear-end accident probability model and can explain the relative changes in rear-end collisions. If the OR value of a variable is greater than 1, the accident probability will increase when the variable value is increased by one unit and the corresponding percentage increase is (OR−1)*100 [41].

## 3. Results

### 3.1. Brake Reaction Time

To quantitatively analyze the effects of mobile distraction tasks and other explanatory variables on the driver’s BRT, a GLMM with an identity link function was used to model the BRT in the LV sudden braking scenario. The driving conditions, all the variables in Table 1, the initial speed (the speed of the SV at the LV brake onset), the ITHW, and the LV deceleration rate were treated as independent variables. In the process of selecting the independent variables, the multicollinearity of the variables was carefully examined to develop the model. The model results in terms of the estimated coefficients, SE (standard error), Z-value, *p* > |z|, and OR for all significant variables are presented in Table 2. The goodness of fit value (chi-squared) for the model was 574.09, and the value of Prob > chi2 was 0.0000 < 0.001. Therefore, the fitted results of the model were acceptable.

(1) $$\begin{array}{cc} BRT & =1.07-0.18*Gender+0.09*Simple\;handheld\;texting+0.15*Complex\;handheld\;texting+0.5*Initial\;time\;headway\\ & -0.03*Initial\;speed+0.21*LV\;deceleration\;\mathrm{rate}\;3m/s^{2}+0.1*LV\;deceleration\;\mathrm{rate}\;5m/s^{2} \end{array}$$

**Figure 3:**
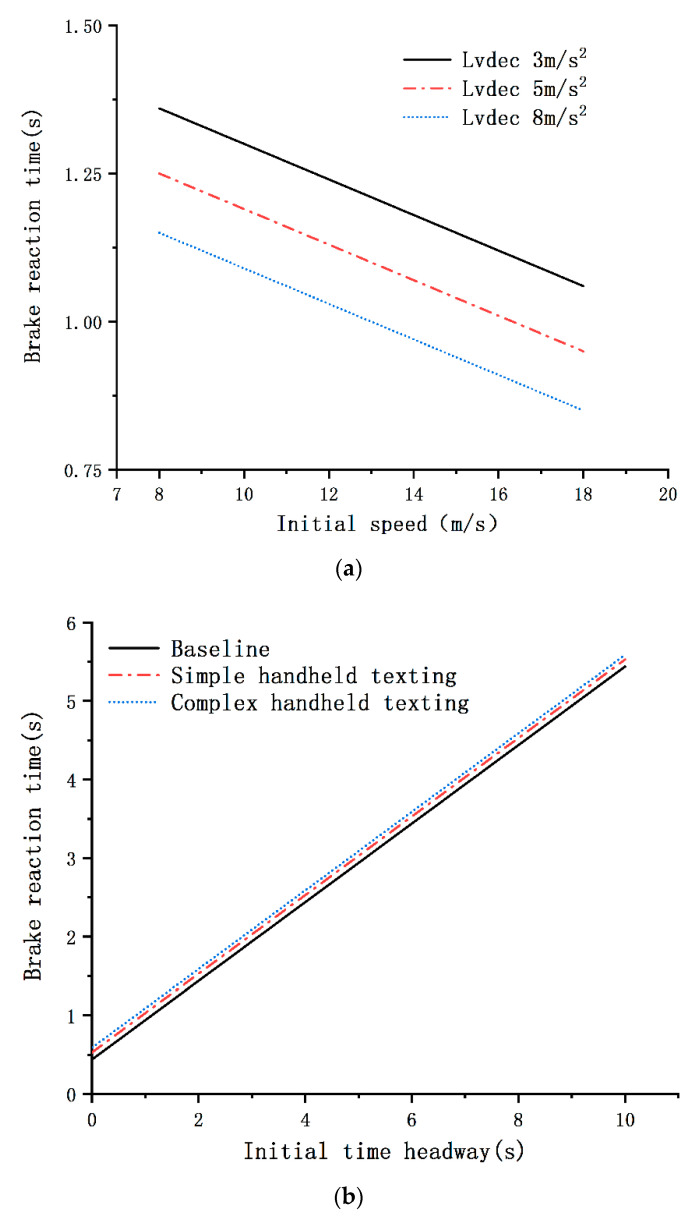

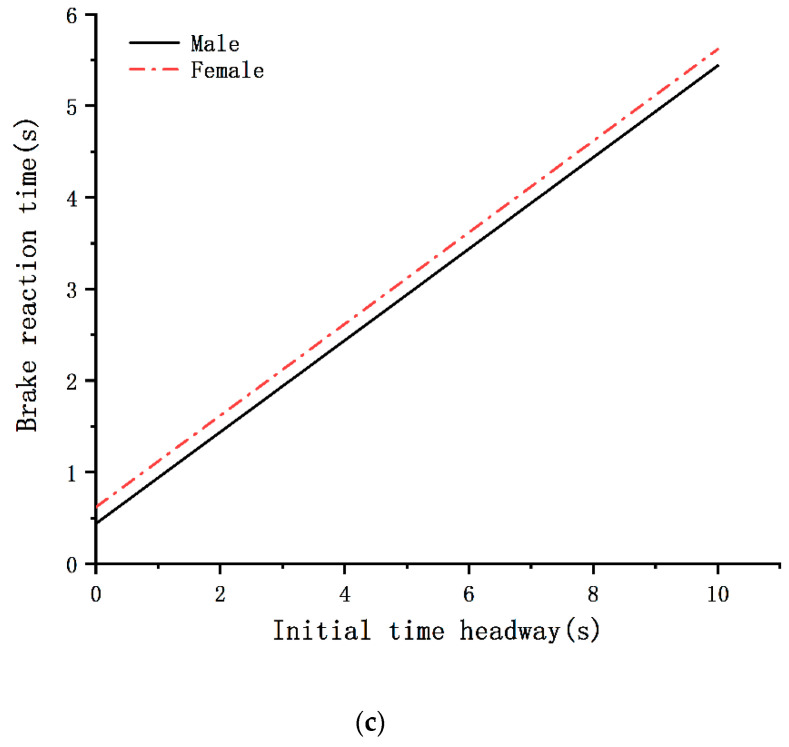
Relationship between the drivers’ brake reaction time (BRT) and the significant variables. (**a**) Non-distracted driving, male, initial time headway (ITHW) = 1 s; (**b**) male, initial speed = 15 m/s, LV deceleration rate = 8 m/s^2^; (**c**) non-distracted driving, initial speed = 15 m/s, LV deceleration rate = 8 m/s^2^.

According to Equation (1), many graphs of the relationship between the drivers’ BRT and the significant variables can be plotted (see Figure 3). The model results exhibited in Table 2 indicate that handheld texting (both simple and complex) had a significant effect on the BRT. However, the effects of speech-based texting (both simple and complex) were not significant. Simple and complex handheld texting respectively resulted in 10% and 17% increases in the BRT as compared to the baseline. Moreover, each 1-s increase in the ITHW led to a 64% increase in the BRT; if the driver’s initial speed was higher, the detection of the LV sudden braking event was 3% faster. In addition to the initial speed and ITHW, the LV braking deceleration rate was also found to have a significant effect on the BRT. Compared to the BRT with the LV deceleration rate of 8 m/s^2^, BRT with the LV deceleration rates of 3 and 5 m/s^2^ were increased by 24% and 11%, respectively. This suggests that the BRT decreased with the increase of the LV deceleration rate. It was also found that male drivers had a shorter BRT than female drivers (see Figure 3c). Interestingly, the effects of the drivers’ driving history and mobile phone use habits proved to be insignificant. 

As can be seen from Figure 3a, the drivers’ BRT was closely related to the initial speed and the LV deceleration rate; it decreased with the increase of the initial speed or the LV deceleration rate. As shown in Figure 3b, at the same initial speed, ITHW, and LV deceleration rate, drivers using a mobile phone while driving (both simple and complex handheld texting) had a greater BRT than those who did not use a mobile phone while driving. Additionally, Figure 3b,c show that the BRT decreased with the decrease of the ITHW.

### 3.2. Rear-End Accident Probability

Because driving behavior characteristics, such as the BRT and ITHW, may play important roles in rear-end collision situations [10,23,24], these variables were also taken as the independent variables while modeling the rear-end accident probability during a sudden braking event. To quantify the impacts of mobile phone distraction and other factors (i.e., BRT, ITHW, LV deceleration rate, driver demographics, driving history, and mobile phone use habits) on rear-end accident probability, a model was developed using the GLMM approach. The GLMM results for rear-end accident probability are presented in Table 3, which includes the estimation coefficients, SE (standard error), Z-value, *p* > |z|, and OR for all significant variables. A chi-squared test was used to check the goodness of fit of the model. The results indicate that the value of Prob > chi2 was 0.0000 < 0.001. Hence, the model results were reasonable.

(2) $$p=\frac{1}{1+e^{-\left(0.85+1.02*Simple\;speech-based\;texting+0.88*Complex\;speech-based\;texting-1.13*Initial\;time\;headway-4.84*LV\;deceleration\;\mathrm{rate}\;3m/s^{2}-2.05*LV\;deceleration\;\mathrm{rate}\;5m/s^{2}\right)}}$$

**Figure 4:**
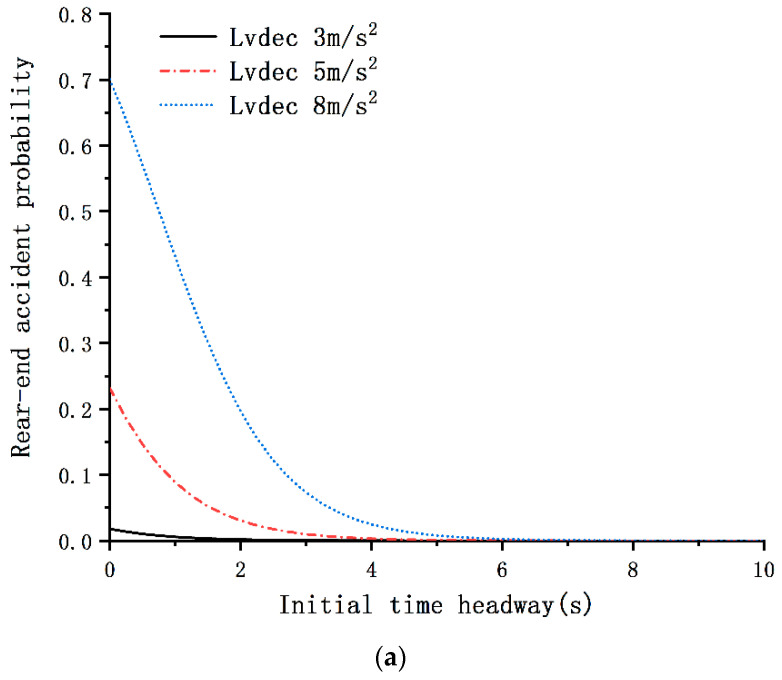

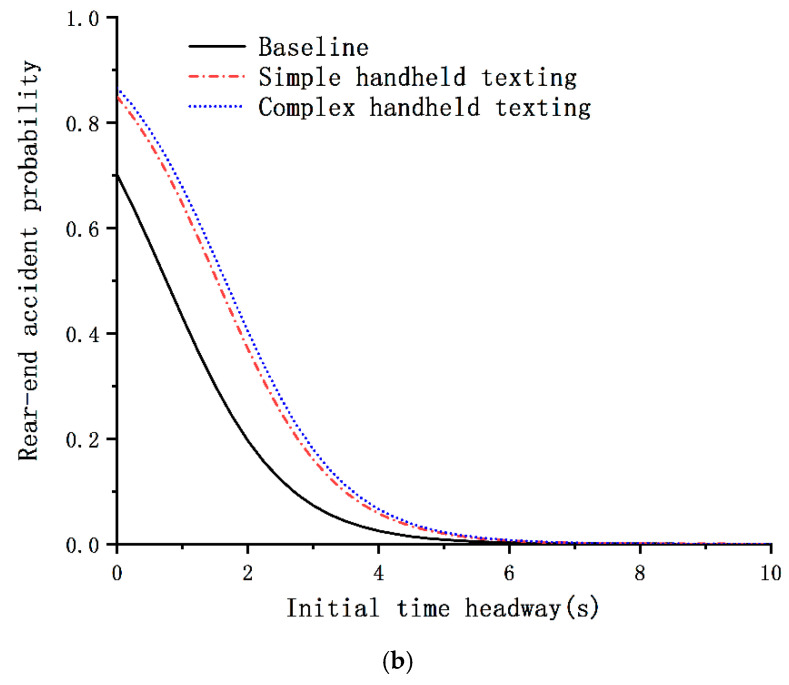
Relationship between rear-end accident probability and the significant variables. (**a**) Non-distracted driving; (**b**) LV deceleration rate = 8 m/s^2^.

The results demonstrate that the accident probability decreased with the increase of the ITHW in the car-following situations (see Figure 4a,b). As shown in Table 3, the rear-end accident probability decreased by 68% with a 1-s increment in the ITHW. Nevertheless, the BRT had no significant effect on the accident probability. Moreover, the LV deceleration rate was found to be positively related to the accident risk; the rear-end accident risk decreased with the decrease of the LV deceleration rate (see Figure 4a). Additionally, as compared to an LV deceleration rate of 8 m/s^2^, the accident probability with the LV deceleration rates of 3 and 5 m/s^2^ was reduced by 99% and 87%, respectively.

According to Equation (2), many probability curves of the relationship between rear-end accident probability and the significant variables can be plotted (see Figure 4). With regard to the distracted driving conditions, all the secondary tasks were expected to result in a high accident risk; surprisingly, however, the results were different. Compared to the baseline (no phone use), it was found that the simple and complex handheld texting tasks increased the rear-end accident probability by 2.41 and 2.77 times, respectively, whereas the speech-based texting tasks (both simple and complex) had no significant effect on accident probability. Figure 4b also illustrates that the accident risk of handheld texting (both simple and complex) was higher than that with no mobile phone use at the same ITHW. Similar to the BRT model, the variables with respect to driving history and mobile phone use habits were considered, but they were found to be insignificant. Interestingly, the driver’s gender was found to have no effect on accident probability.

### 3.3. Effects of LV Deceleration Rate and Actual Driving Behavior Characteristics on the Observed Rear-End Accident Risk

**Figure 5:**
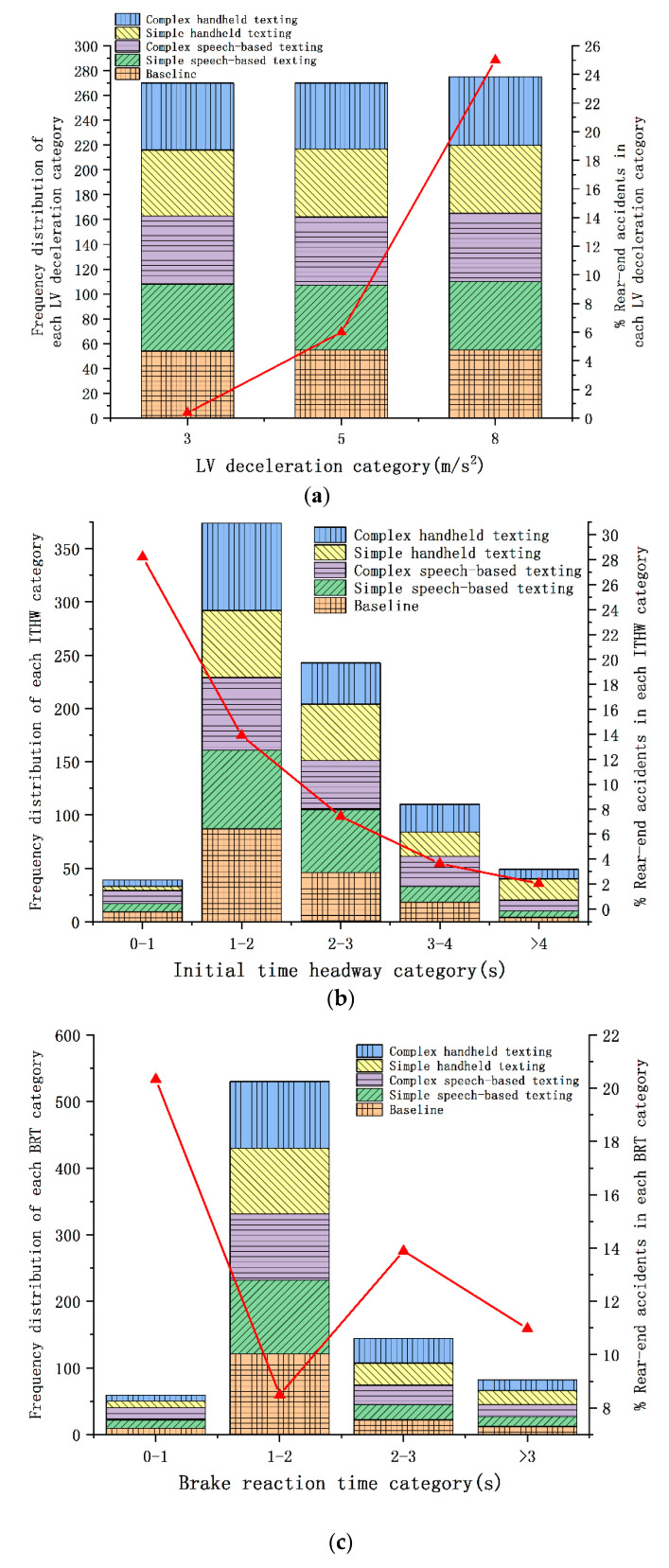
Frequency distributions of BRT, ITHW, and LV deceleration categories with the percentage of observed accidents in different driving conditions. (**a**) LV deceleration category; (**b**) ITHW category; (**c**) BRT category.

The effects of mobile phone distraction tasks, the LV deceleration rate, and the actual driving behavior characteristics (i.e., BRT and ITHW) on the observed rear-end accidents in an emergency braking scenario are presented in Figure 5.

To investigate the impacts of the driving conditions and three levels of LV deceleration (3, 5, and 8 m/s^2^) on the observed accident risk, the frequencies of the observed driving conditions of each LV deceleration category are shown in Figure 5a (see left *Y*-axis), and the observed percentage of accident frequency in each LV deceleration category is presented in Figure 5a (see right *Y*-axis). The frequency percentage of observed accidents (represented as a red triangle symbol) was estimated by the ratio of the frequency of accidents observed in each LV deceleration category to the frequency of all driving conditions in this category.

Similarly, to explore the effects of mobile phone distracted driving conditions and ITHW on the observed accidents, the frequency distribution of the ITHW of all participants was classified into five categories: 0–1, 1–2, 2–3, 3–4, and >4 s. The frequency distribution of the ITHW for each category is presented in Figure 5b (see left *Y*-axis). For each ITHW category, the frequencies of the five driving conditions were observed to explore the contribution of each driving condition. Further, the percentage of accident frequencies observed in each category is represented by the right *Y*-axis in Figure 5b (represented as a red triangle symbol). The accident frequency percentage was estimated as the ratio of the frequency of accidents observed in each ITHW category to the frequency of all participants in this category. Similar to Figure 5b, the effects of the BRT and five driving conditions on the observed accidents are presented in Figure 5c; the observed BRT was divided into four categories: 0–1, 1–2, 2–3, and >3 s.

For a sudden emergency braking event in an urban road car-following scene, Figure 5a reveals that the increase of the LV deceleration rate led to more rear-end accidents; rear-end accidents were therefore found to be closely related to the level of LV deceleration. In addition, it can also be observed that there were very few rear-end accidents in the category with a LV deceleration of 3 m/s^2^, which means that the driver could almost control the driving safety of the vehicle when the LV deceleration rate was small.

Figure 5b indicates that a shorter ITHW resulted in a higher risk of accident during the LV sudden braking event. The highest observed frequency percentage of rear-end collisions was 28% in the shortest ITHW category (i.e., 0–1 s). Compared with handheld texting (both simple and complex tasks), it can also be found that the baseline and speech-based texting (both simple and complex tasks) contributed the most to the frequency of the shortest ITHW category. Furthermore, the ITHW frequency distribution indicates that most of the observations from the baseline and speech-based texting fell within the short ITHW range. Hence, the occurrence of rear-end accidents in the baseline and speech-based texting (i.e., simple and complex tasks) conditions was mainly caused by the short ITHW. However, rear-end accidents that occurred during simple and complex handheld texting tasks were due to the effects of a short ITHW and attention shifting from the primary driving task to the secondary tasks. In the longest initial time headway category (i.e., ITHW > 4 s), handheld texting (both simple and complex tasks) had the highest contribution to the frequency.

As can be seen from Figure 5c, the relationship between the observed accident frequency percentage and BRT did not follow a specific pattern. Moreover, the highest percentage of observed rear-end accidents was in the fastest BRT category (i.e., 0–1 s), suggesting that there may be other potential factors that affected the accident probability. For example, the increase of the LV deceleration rate and the decrease of the ITHW may have been the main reasons for the high accident probability. The lowest percentage of rear-end accidents observed was 8% in the 1–2 s brake reaction time category. It can also be observed that the frequency of all driving conditions was also mainly distributed in this category.

## 4. Discussion

The aim of this research was to analyze and compare the effects of mobile phone distraction caused by speech-based and handheld texting on rear-end accident risk during a sudden braking event in a Chinese urban road environment. The study was conducted using 55 young Chinese drivers under urban road driving conditions on a driving simulator. Their driving performance metrics were modeled in terms of the BRT and rear-end accident probability.

The results of the GLMM for BRT showed that handheld texting tasks (both simple and complex) resulted in delayed responses to a sudden braking event as compared to non-distracted driving. However, receiving and sending speech-based texts (both simple and complex) via WeChat software resulted in no delay in the response to sudden braking events. This may be due to the fact that speech-based texting tasks involved less visual distraction compared to hand-held texting tasks. An interesting finding of this study was that the difficulty level of handheld texting tasks affected the drivers’ BRT; complex handheld texting tasks increased the BRT in response to the sudden braking event as compared to simple handheld texting. This may be attributed to the fact that the share of increased attention was assigned to the secondary tasks rather than driving to handle the increased level of complexity in the handheld texting tasks. Similar to previous research results [10], the results of this study show that male drivers responded faster to sudden braking events than female drivers in a car-following scenario (see Figure 3c). Another finding of this study was that the drivers’ BRT decreased when the LV deceleration rate increased, when the initial speed increased, or when the ITHW decreased. This result is consistent with the results of a previous study conducted by Wang et al. [2], who found that drivers are more alert and respond faster when the LV deceleration rate increases or the ITHW decreases. This is likely attributed to the fact that drivers pay more attention to driving tasks when they are driving at high rear-end-collision urgency levels.

The results of the present study revealed that the driving behavior characteristics of young drivers and the behavior of the LV have significant impacts on accident risk; a shorter ITHW and faster LV deceleration rate will lead to a higher accident risk. Although the drivers’ BRT decreased as the ITHW decreased and the LV deceleration rate increased, the rear-end accident risk still increased. This result suggests that the reduction in reaction time is not sufficient to compensate for the increased accident risk. The ORs of the results indicated that a 1-s decrease in the ITHW led to an increment of the rear-end accident probability by 3.13 times. However, the effect of the BRT was not found to be significant. These results may imply that, even without any distraction task, the probability of accidents will increase by many times for drivers with a shorter ITHW during a sudden braking event.

Compared with the baseline driving condition, the results showed that simple and complex handheld texting while driving increased the rear-end accident probability by 2.41 and 2.77 times, respectively. Surprisingly, the accident probability was not significantly increased for young drivers when they were involved in speech-based texting tasks while driving. However, drivers engaging in speech-based texting tasks while driving may also have a higher accident risk if they are driving the vehicle at a short ITHW and if the LV deceleration rate is fast. The best advice for drivers is to avoid all distractions, including speech-based and handheld texting tasks, and to focus only on driving tasks. One false belief is that speech-based texting is safe for driving, which may increase exposure to this secondary task and consequently overshadow the potential benefits of speech-based texting over handheld texting, thus ultimately leading to an increased number of traffic accidents.

Accuracy is a key performance indicator for potential accident risk prediction [42,43]. Prediction with low accuracy means that a forward collision warning system will sound an alarm too early or too late. A late warning means that drivers will not have enough time to react to the approaching hazards, which may lead to more collisions than if the system was not used. On the other hand, the earlier the warning, the more likely it is to be considered a false alarm, which would reduce drivers’ trust in the forward collision warning system and the long-term use of the system [44]. Therefore, to improve the accuracy and acceptance of a forward collision warning system, the accuracy of potential risk prediction is crucial. The traditional forward collision warning system predicts the potential risk based on vehicle performance data (e.g., speed, time headway, time to collision, etc.) that is continuously collected from radars or visual sensors [45,46]. However, the results of this study suggest that the quantitative effects of mobile phone distracted driving conditions, the driving behavior characteristics of drivers, and the LV deceleration rate should be considered for the prediction of potential risk.

## 5. Conclusions

Using mobile phones while driving has become one of the main causes of traffic accidents and poses a serious threat to public health. To investigate the impacts of speech-based texting and handheld texting on rear-end accident risk and to identify potential predictors of rear-end accidents, especially during sudden braking events, a GLMM method was developed to statistically model and quantify the effects of the driving behavior characteristics of young drivers, the behavior of the LV, and mobile phone distraction tasks (i.e., both speech-based and handheld texting) on accident risk associated with a sudden braking event. The results showed that handheld texting tasks (both simple and complex) resulted in delayed response to sudden braking events and increased rear-end accident risk compared to the baseline. In addition, the use of speech-based texting tasks while driving had less impact on the rear-end accident risk than handheld texting tasks. The results also showed that the rear-end accident risk increased if the driver drove the vehicle with a shorter initial time headway or a higher LV deceleration rate.

Therefore, these results raise serious questions about the appropriateness of the current legislation on the use of mobile phones during driving and can provide theoretical guidance for legislative bodies to formulate traffic laws on mobile phone use while driving. Additionally, the current study results can help the regulatory bodies to promote public awareness about the serious negative effects of using a mobile phone while driving. More importantly, these results suggest that an algorithm designed for a forward collision warning system should consider the effects of mobile phone distraction tasks, the behavior of the LV, and the driving behavior characteristics. 

However, there were some limitations of this study that must be acknowledged. The results of the present study were based on a driving simulator, which may produce different results from real-world driving; thus, the reported results require further verification by a field study. Another limitation is that the only participants in the study were young drivers with less driving experience. Therefore, the sample in future research should be expanded to include middle-aged and older drivers to generalize the results for all age categories. Moreover, future research should examine the difference in exposure levels of texting (speech-based and handheld) while driving. The speed of the LV should also be an important factor that may affect the driving behavior characteristics of drivers, although this variable was kept constant in the present study. Therefore, different LV speeds should be investigated as influential factors in rear-end accident risk models in future research. Finally, in this study, there was no traffic flow in the adjacent lane of the SV. However, in a real driving environment, the driver must interact with the surrounding traffic; hence, the impact of scene complexity on driving behavior should be further explored. 

## Figures and Tables

**Table 1:** Statistical description of the participants obtained from the questionnaire.

Variable	Percentage	Mean	SD
Driver demographics			
Age		25.13	2.57
Gender			
Male	72.73		
Female *	27.27		
Driving history			
Years of driving		3.05	2.31
Kilometers driven			
0–5000 km *	78.18		
5000–10,000 km	10.91		
>10,000 km	10.91		
Traffic fines due to mobile phone use in the last three years			
None *	100		
Once	0		
More than once	0		
Crash involvement history in the last three years			
None *	94.54		
Once	3.64		
More than once	1.82		
Traffic accidents due to mobile phone use in the last three years			
None *	100		
Once	0		
More than once	0		
Mobile phone use habits			
Frequency of speech-based texting use while driving			
None or less *	36.36		
Sometimes	41.82		
Frequently	21.82		
Frequency of handheld texting use while driving			
None or less *	58.18		
Sometimes	38.18		
Frequently	3.64		

Note: Cat = categorical variable; Con = continuous variable; SD = standard deviation; * reference category.

**Table 2:** Results of the generalized linear mixed model for the brake reaction time.

Parameter	Estimate	SE	z	*p* > |z|	OR
Intercept	1.07	0.26	4.05	<0.001	2.92
Gender (Female *)	−0.18	0.10	−1.85	0.065	0.84
Simple speech-based texting (No phone *)	0.07	0.05	1.28	0.201	1.07
Complex speech-based texting (No phone *)	0.04	0.05	0.72	0.469	1.04
Simple handheld texting (No phone *)	0.09	0.05	1.71	0.087	1.10
Complex handheld texting (No phone *)	0.15	0.05	2.89	0.004	1.17
Initial time headway	0.50	0.02	21.89	<0.001	1.64
Initial speed	−0.03	0.02	−1.82	0.069	0.97
LV deceleration rate 3 m/s^2^ (LV deceleration rate 8 m/s^2^ *)	0.21	0.04	5.29	<0.001	1.24
LV deceleration rate 5 m/s^2^ (LV deceleration rate 8 m/s^2^ *)	0.10	0.04	2.56	0.011	1.11
Goodness of fit	Wald chi2(9) = 574.09, Prob > chi2 = 0.0000 < 0.001				

Note: SE: standard error; OR: odds ratio; * reference category.

**Table 3:** Model results for rear-end accident probability during a sudden braking event.

Parameter	Estimate	SE	z	*p* > |z|	OR
Intercept	0.85	0.22	3.86	<0.001	2.34
Simple speech-based texting (No phone *)	0.61	0.45	1.36	0.174	1.84
Complex speech-based texting (No phone *)	0.45	0.46	0.98	0.327	1.57
Simple handheld texting (No phone *)	0.88	0.44	2	0.045	2.41
Complex handheld texting (No phone *)	1.02	0.45	2.28	0.023	2.77
Initial time headway	−1.13	0.22	−5.25	<0.001	0.32
LV deceleration rate 3 m/s^2^ (LV deceleration rate 8 m/s^2^ *)	−4.84	1.02	−4.74	<0.001	0.01
LV deceleration rate 5 m/s^2^ (LV deceleration rate 8 m/s^2^ *)	−2.05	0.33	−6.25	<0.001	0.13
Goodness of fit	Wald chi2(8) = 74.49, Prob > chi2 = 0.0000 < 0.001				

Note: SE: standard error; OR: odds ratio; * reference category.

## References

[1] World Health Organization (2020). Road Traffic Injuries. https://www.who.int/en/news-room/fact-sheets/detail/road-traffic-injuries.

[2] Wang X., Zhu M., Chen M., Tremont P. (2016). Drivers’ rear end collision avoidance behaviors under different levels of situational urgency. Transp. Res. Part C Emerg. Technol..

[3] U.S. Department of Transportation, National Highway Traffic Safety Administration (2017). By First Harmful Event, Type of Collision and Crash Severity. https://www.iii.org/fact-statistic/facts-statistics-highway-safety#Crashes.

[4] National Center for Statistics and Analysis (2019). Distracted Driving in Fatal Crashes, 2017.

[5] World Health Organization (2018). Global Status Report on Road Safety 2018.

[6] Oviedo-Trespalacios O. (2018). Getting away with texting: Behavioural adaptation of drivers engaging in visual-manual tasks while driving. Transp. Res. Part A Policy Pract..

[7] Shi J., Xiao Y., Atchley P. (2016). Analysis of factors affecting drivers’ choice to engage with a mobile phone while driving in Beijing. Transp. Res. Part F Traffic Psychol. Behav..

[8] World Health Organization (2011). Mobile Phone Use: A Growing Problem of Driver Distraction.

[9] Choudhary P., Velaga N.R. (2018). Performance degradation during sudden hazardous events: A comparative analysis of use of a phone and a music player during driving. IEEE Trans. Intell. Transp. Syst..

[10] Li X., Yan X., Wu J., Radwan E., Zhang Y. (2016). A rear-end collision risk assessment model based on drivers’ collision avoidance process under influences of cell phone use and gender—A driving simulator based study. Accid. Anal. Prev..

[11] Zhang H., Qian D., Yang X., Shao C. (2019). The impact of speech-based texting on drivers’ braking reaction and deceleration duration in the car-following scenario. J. Transp. Saf. Secur..

[12] Calvi A., Benedetto A., D’Amico F. (2018). Investigating driver reaction time and speed during mobile phone conversations with a lead vehicle in front: A driving simulator comprehensive study. J. Transp. Saf. Secur..

[13] Drews F.A., Yazdani H., Godfrey C.N., Cooper J.M., Strayer D.L. (2009). Text messaging during simulated driving. J. Hum. Factors Ergon. Soc..

[14] Yannis G., Laiou A., Papantoniou P., Christoforou C. (2014). Impact of texting on young drivers’ behavior and safety on urban and rural roads through a simulation experiment. J. Saf. Res..

[15] Haque M., Washington S. (2013). Effects of mobile phone distraction on drivers’ reaction times. J. Australas. Coll. Road Saf..

[16] He J., Chaparro A., Nguyen B., Burge R.J., Crandall J., Chaparro B., Ni R., Cao S. (2014). Texting while driving: Is speech-based text entry less risky than handheld text entry. Accid. Anal. Prev..

[17] Yager C. (2013). An Evaluation of the Effectiveness of Voice-to-Text. Programs at Reducing Incidences of Distracted Driving.

[18] Atwood J., Guo F., Fitch G., Dingus T.A. (2018). The driver-level crash risk associated with daily cellphone use and cellphone use while driving. Accid. Anal. Prev..

[19] Choudhary P., Velaga N.R. (2017). Mobile phone use during driving: Effects on speed and effectiveness of driver compensatory behaviour. Accid. Anal. Prev..

[20] Caird J.K., Johnston K.A., Willness C.R., Asbridge M., Steel P. (2014). A meta-analysis of the effects of texting on driving. Accid. Anal. Prev..

[21] Klauer S.G., Guo F., Simons-Morton B.G., Ouimet M.C., Lee S.E., Dingus T.A. (2014). Distracted driving and risk of road crashes among novice and experienced drivers. N. Engl. J. Med..

[22] Li X., Oviedo-Trespalacios O., Rakotonirainy A., Yan X. (2019). Collision risk management of cognitively distracted drivers in a car-following situation. Transp. Res. Part F Traffic Psychol. Behav..

[23] Winsum W.V., Heino A. (1996). Choice of time-headway in car-following and the role of time-to-collision information in braking. Ergonomics.

[24] Chen Y., Fu R., Xu Q., Yuan W. (2020). Mobile Phone Use in a Car-Following Situation: Impact on Time Headway and Effectiveness of Driver’s Rear-End Risk Compensation Behavior via a Driving Simulator Study. Int. J. Environ. Res. Public Health.

[25] Vogel K. (2003). A comparison of headway and time to collision as safety indicators. Accid. Anal. Prev..

[26] Hosking S.G., Young K.L., Regan M.A. (2009). The effects of text messaging on young drivers. Hum. Factors.

[27] Jamson A.H., Westerman S.J., Hockey G.R.J., Carsten O.M. (2004). Speech-based e-mail and driver behavior: Effects of an in-vehicle message system interface. Hum. Factors.

[28] Saifuzzaman M., Haque M.M., Zheng Z., Washington S. (2015). Impact of mobile phone use on car-following behaviour of young drivers. Accid. Anal. Prev..

[29] Lee J.D., McGehee D.V., Brown T.L., Reyes M.L. (2002). Collision warning timing, driver distraction, and driver response to imminent rear-end collisions in a high-fidelity driving simulator. Hum. Factors.

[30] Haque M.M., Washington S. (2014). A parametric duration model of the reaction times of drivers distracted by mobile phone conversations. Accid. Anal. Prev..

[31] Törnros J., Bolling A. (2006). Mobile phone use–effects of conversation on mental workload and driving speed in rural and urban environments. Transp. Res. Part F Traffic Psychol. Behav..

[32] Metz B., Landau A., Hargutt V. (2015). Frequency and impact of hands-free telephoning while driving–Results from naturalistic driving data. Transp. Res. Part F Traffic Psychol. Behav..

[33] Fitch G.M., Soccolich S.A., Guo F., McClafferty J., Fang Y., Olson R.L., Dingus T.A. (2013). The Impact of Hand-Held and Hands-Free Cell Phone Use on Driving Performance and Safety-Critical Event Risk.

[34] Scott-Parker B., Oviedo-Trespalacios O. (2017). Young driver risky behaviour and predictors of crash risk in Australia, New Zealand and Colombia: Same but different. Accid. Anal. Prev..

[35] Bendak S., Alali A.K., Alali N.M., Alshehhi M.M. (2019). Is the use of mobile phones while driving reaching alarming rates? A case study. Transp. Lett..

[36] Tucker S., Pek S., Morrish J., Ruf M. (2015). Prevalence of texting while driving and other risky driving behaviors among young people in Ontario, Canada: Evidence from 2012 and 2014. Accid. Anal. Prev..

[37] Cook J.L., Jones R.M. (2011). Texting and accessing the web while driving: Traffic citations and crashes among young adult drivers. Traffic Inj. Prev..

[38] Thapa R., Codjoe J., Ishak S., Mccarter K.S. (2015). Post and during event effect of cell phone talking and texting on driving performance—a driving simulator study. J. Crash Prev. Inj. Control.

[39] Islam M.R., Wyman A.A., Hurwitz D.S. (2017). Safer driver responses at intersections with green signal countdown timers. Transp. Res. Part F Traffic Psychol. Behav..

[40] Oviedo-Trespalacios O., Haque M.M., King M., Washington S. (2017). Effects of road infrastructure and traffic complexity in speed adaptation behaviour of distracted drivers. Accid. Anal. Prev..

[41] Yan X., Radwan E., Abdel-Aty M. (2005). Characteristics of rear-end accidents at signalized intersections using multiple logistic regression model. Accid. Anal. Prev..

[42] Ba Y., Zhang W., Wang Q., Zhou R., Ren C. (2017). Crash prediction with behavioral and physiological features for advanced vehicle collision avoidance system. Transp. Res. Part C Emerg. Technol..

[43] Wu C., Peng L., Huang Z., Zhong M., Chu D. (2014). A method of vehicle motion prediction and collision risk assessment with a simulated vehicular cyber physical system. Transp. Res. Part C Emerg. Technol..

[44] Jamson A.H., Lai F.C., Carsten O.M. (2008). Potential benefits of an adaptive forward collision warning system. Transp. Res. Part C Emerg. Technol..

[45] Wang J., Yu C., Li S.E., Wang L. (2016). A forward collision warning algorithm with adaptation to driver behaviors. IEEE Trans. Intell. Transp. Syst..

[46] Xiong X., Wang M., Cai Y., Chen L., Farah H., Hagenzieker M. (2019). A forward collision avoidance algorithm based on driver braking behavior. Accid. Anal. Prev..

